# DACH1 antagonizes CXCL8 to repress tumorigenesis of lung adenocarcinoma and improve prognosis

**DOI:** 10.1186/s13045-018-0597-1

**Published:** 2018-04-10

**Authors:** Qian Liu, Anping Li, Shengnan Yu, Shuang Qin, Na Han, Richard G. Pestell, Xinwei Han, Kongming Wu

**Affiliations:** 1 Department of Oncology, Tongji Hospital of Tongji Medical College, Huazhong University of Science and Technology, Wuhan, 430030 People’s Republic of China; 2 Department of Interventional Radiology, The First Affiliated Hospital of Zhengzhou University, Zhengzhou, 450052 People’s Republic of China; 3 Pennsylvania Cancer and Regenerative Medicine Research Center, Wynnewood, PA 19096 USA

**Keywords:** CXCL8, DACH1, Lung cancer, Progression, Prognosis, AP-1, NF-κB

## Abstract

**Background:**

C-X-C motif ligand 8 (CXCL8), known as a proinflammatory chemokine, exerts multiple effects on the proliferation, invasion, and migration of tumor cells via the autocrine or paracrine manner. Conversely, the human Dachshund homologue 1 (DACH1) is recognized as a tumor suppressor which retards the progression of various cancers. In prostate cancer, it has been demonstrated that DACH1 was negatively correlated with the expression of CXCL8 and able to antagonize the effects of CXCL8 on cellular migration. Herein, we explored the mechanisms by which DACH1 regulated the CXCL8 in non-small cell lung cancer (NSCLC).

**Methods:**

Public microarray and Kaplan-Meier plotter datasets were analyzed. Blood serum samples from lung adenocarcinoma (ADC) patients were collected for enzyme-linked immunosorbent assay (ELISA) analysis. Immunohistochemical staining was conducted on tissue microarray. Cell lines with stable expression of DACH1 were established, and relative gene expression was measured by Western blot, ELISA, real-time PCR, and human cytokine array. Correspondingly, cell lines transfected with shDACH1 were established, and relative gene expression was measured by real-time PCR and immunofluorescence array. Functional studies were performed by transwell and xenograft mice models. Luciferase reporter gene assay was applied to measure the regulation of DACH1 on CXCL8.

**Results:**

Our study indicated that CXCL8 both at the mRNA and protein level was associated with the high tumor burden of ADC. Correlational analyses in ADC cell lines and ADC tissues showed that DACH1 was inversely correlated with CXCL8. Meanwhile, patients with high DACH1 expression and low CXCL8 expression had prolonged time to death and recurrence. Moreover, we verified the inhibitory effects of DACH1 on CXCL8 both in vitro and in vivo. Mechanism studies proved that DACH1 transcriptionally repressed CXCL8 promoter activity through activator protein-1 (AP-1) and nuclear transcription factor-kappa B (NF-κB) sites.

**Conclusions:**

Our study proved that CXCL8 acted as an unfavorable factor promoting to tumor progression and poor prognosis of ADC, while DACH1 antagonized CXCL8 to provide a favorable survival of ADC patients. Double detection of DACH1 and CXCL8 may provide a precise information for further evaluating the prognosis of ADC patients.

## Background

CXCL8, also known as interleukin-8 (IL-8), belongs to glutamic acid-leucine-arginine (ELR)^+^ CXC chemokines. It is generally produced by macrophages, epithelial cells, airway smooth muscle cells, and endothelial cells [1]. CXCL8 is rapidly induced by proinflammatory cytokines such as tumor necrosis factor-α (TNF-α) and interleukin-1β (IL-1β) [2]. Similar to other inflammatory factors, such as CXCL5 and IL-6, CXCL8 is responsible for the recruitment and activation of neutrophils and granulocytes to the site of inflammation [3–6]. Inflammatory responses with great number of cytokines in tumor microenvironment play more prominent roles in promoting tumor growth, progression, and immunosuppression than launching an effective antitumor response [7]. In the context of cancer, CXCL8 acts as an important multifunctional cytokine to modulate proliferation, invasion, and migration of tumor cells via an autocrine or paracrine manner. Lung cancer cell lines were shown to express increased CXCL8 which promotes tumor growth, angiogenesis, and invasion [8, 9]. Findings thus far suggested that overexpressed CXCL8 played a vital role in initiation and progression of lung adenocarcinoma (ADC) and presented early relapse and unfavorable prognosis of patients [10].

The *Dachshund* gene was initially cloned as a dominant inhibitor of the hyperactive epidermal growth factor receptor (EGFR), ellipse, in drosophila [11]. DACH1 acts as a regulator for expressions of targeted genes through binding to specific DNA sequences directly or interacting with other transcription factors (c-Jun, Smads, Six, and ER-α) [12, 13]. Previous studies suggested that DACH1 was required for the development of some tissues, and abnormal expression of DACH1 was related with several diseases [13, 14]. Several lines of evidence showed that DACH1 may represent a novel tumor repressor [13, 15], which inhibited growth and metastasis of breast cancer in vivo via blocking Wnt [15]; transforming growth factor-beta (TGF-β) [16], cyclin D1 [17], and estrogen receptor-alpha (ER-α) [18] signaling; and interfering breast tumor stem cell function [19]. The loss of DACH1, which induces proliferation and invasion of tumor cells, has been observed in prostate cancer, endometrial cancer, renal cancer, and gastric cancer [20–22]. Recently, reduced DACH1 was observed in ADC at both mRNA and protein level [23]. DACH1 blocked the growth of ADC cells and enhanced cell-cycle arrest in a p53-dependent manner [23]. In addition, DACH1 inhibited peroxiredoxin 3 (PRX3)-mediated tumorigenesis and invasion of ADC [24].

CXCL8 was identified as a downstream target of DACH1 in the process of suppression of cell proliferation and migration in breast and prostate cancer [20, 21]. Recent study reported that DACH1 inhibited growth and metastasis of ADC by repressing the expression of CXCL5 [25]. However, the relationship between DACH1 and CXCL8 in ADC is not explicit. In this study, we showed that expression of CXCL8, which was closely associated with clinic-pathological features and clinical outcome of ADC at both mRNA and protein level, was controlled by DACH1 in vitro and in vivo. Furthermore, the relative expressions of CXCL8 and DACH1 determine the further prognosis of ADC patients.

## Methods

### Microarray analysis

CXCL8 mRNA expression datasets for lung cancer were downloaded from the ArrayExpress. GSE31210 expression profile consists of 226 primary ADC patients with pathological stage I–II. GSE68465 with 443 ADC cases includes histologic grade and tumor size. GSE32474 consists of nine lung cancer cell lines. Meta-analysis integrating 10 studies was performed as previously described [26], and the forest graphs were drawn by Stata 13.

### Cell culture, establishment of cell lines stably expressing DACH1 and cell stimulation

The 293T and human bronchial epithelial (HBE) cell lines were cultured in Dulbecco’s modified Eagle’s medium (DMEM) supplemented with 10% fetal bovine serum (FBS), and human lung cancer cell lines (SKLU, A549, and H460) were cultured in 1640 medium supplemented with 10% FBS. Cells were maintained in an atmosphere of 5% carbon dioxide (CO_2_) in a humidified 37 °C incubator. The expression vectors encoding DACH1 and DACH1 deficient in the DNA binding domain (ΔDS) were subcloned into lentivirus expression vector. Stable expressions of DACH1 in A549 and SKLU cells were confirmed by immunofluorescence. In the drug intervention group, 100 ng/ml 12-O-tetradecanoyl phorbol-13-acetate (TPA) and 10 ng/ml TNF-α were added into the supernatants, respectively, after 24 h of serum starvation. After 12 h of the stimulation, cells were subsequently used for further experiments.

### ELISA

The blood samples were collected from about 100 patients who were diagnosed as ADC at various stages by April 2015 in Tongji hospital. The QuantiCyto Hu CXCL8 kit (H161125-008a) is a solid phase sandwich ELISA kit, used for the quantitative determination of CXCL8 in human serum, plasma, buffered solution, or cell culture medium. A monoclonal antibody specific for CXCL8 has been coated onto the wells of the microtiter strips provided by the manufacture. Diluted samples, including standards of known CXCL8 content, control specimens, and unknowns, were pipetted into these wells followed by the addition of a second biotinylated monoclonal antibody. Then, Streptavidin-Peroxidase (enzyme) was added to complete the four-member sandwich. After incubation and washing steps to rid the microplate of unbound substances, a substrate solution (TMB) was added and reacted with the enzyme-antibody-target complex to produce measurable signals. After stopping the reaction with the stop reagent, the values of optical densities (OD) were measured at 450 nm using Microplate Reader (BioRad). The intensity of this signal is directly proportional to the concentration of CXCL8 in original specimen.

### Immunohistochemical staining

Commercially available tissue microarray slide (HLug-Ade150Sur-02, Shanghai Outdo Biotech Company) with 75 pairs of ADC samples and corresponding adjacent tissues was used to assess the correlation between the protein expression of CXCL8 and clinic-pathological variables and prognosis of ADC patients based on the detailed survival data. Specific primary antibody of CXCL8 (RLT2343, Ruiying Biology, 1:100) was utilized for immunohistochemistry (IHC) with a two-step protocol which was described formerly [27, 28]. Additionally, the protein expressions of DACH1, cyclin D1, Ki-67, and CXCL8 in A549-vector and A549-DACH1 xenograft mice models were examined by IHC. The specific primary antibodies were anti-DACH1-antibody (10914-1-AP, ProteinTech, 1:150), anti-Ki-67-antibody (ab15680, Abcam, 1:150), and anti-CXCL8-antibody (RLT2343, Ruiying Biology, 1:250). Ready to use rabbit anti-cyclin D1-antibody was from MAIXIN-Bio (RMA-0541). Slide images were captured by Mv Image software.

### Analysis and quantification of staining

The immunohistochemical scores were graded by two people independently. Scores were dependent on the intensity and percentage of positive staining tumor cells in the whole tissue staining according to the Fromowitz standard as described above [29]. The staining intensity was scored as 0 (no staining), 1 (weak staining), 2 (moderate staining), and 3 (strong staining). The percentage of positive cells was divided into four levels: 1 (0–25% positive cells), 2 (26–50% positive cells), 3 (51–75% positive cells), and 4 (76–100% positive cells). The multiplication of the intensity and percentage was utilized to represent the final staining score. Score > 8 was defined as high CXCL8 staining, otherwise was defined as low CXCL8 staining. Moreover, at least five fields of × 200 magnification from each core were taken for quantification.

### Human cytokine array

Human cytokine arrays spotted on nitrocellulose membranes were obtained from Raybiotech (Norcross, GA). Conditioned medium was prepared from SKLU-vector, SKLU-DACH1, A549-vector, and A549-DACH1 cells by culturing cells in serum free 1640 for 24–48 h. Cytokine array analysis was performed as described previously [21].

### Western blot analysis

Cells were washed twice with cold phosphate buffered solution (PBS) and then lysed with RIPA lysis buffer containing 1 mM phenylmethanesulfonyl fluoride (PMSF). Each sample was denatured in 100 °C boiling water in the presence of sodium dodecyl sulfate (SDS) and DL-Dithiothreitol (DTT). Twenty micrograms of protein from each sample was loaded on a 15% SDS-polyacrylamide gel, and the separated proteins were then transferred onto a polyvinylidene fluoride (PVDF) membrane. The primary antibodies were anti-CXCL8-antibody (60141-2-lg, ProteinTech, 1:1000), anti-DACH1-antibody (10914-1-AP, ProteinTech, 1:1000), and anti-β-actin-antibody (60008-1-lg, ProteinTech, 1:2000). Secondary staining and detection were conducted in accordance with standard protocols as previously described [30, 31].

### Real-time PCR analysis

The primers of CXCL8 used for real-time PCR are as follows: forward primer, 5′- GACAGCAGAGCACACAAGC-3′; reverse primer, 5′- GGCAAAACTGCACCTTCAC-3′, which were applied in previous study [32]. Total mRNA was extracted from cells using Trizol on the basis of the manufacturer’s protocol (Takara Co, Japan). One microgram of total mRNA was subsequently reverse transcribed to complementary DNA (cDNA) with synthesis kit (TransStart). Quantification reactions were carried out using the ABI 7900 HT platform. Each reaction system was composed of 3 μl cDNA, 5 μl Tip Green qPCR SuperMix (TransStart), 2 μl 10 μM forward and reverse primer mix. Samples were amplified under the following cycling conditions: 95 °C for 30 s, and 45 cycles of 95 °C for 5 s, 59 °C for 15 s, and 72 °C for 10 s. Cycle thresholds were calculated as the expression fold changes through formula: 2^-△△CT^.

### Transwell migration assay

Transwell migration assays were conducted using an 8-μm pore size transwell filter insert (Corning Inc., Corning, NY, USA) coated with diluted Matrigel (BD Biosciences, Bedford, MA, USA). SKLU-vector, SKLU-DACH1, A549-vector, and A549-DACH1 cells were suspended in 1640 culture medium with 1% FBS, and 2.5 × 10^4^ cells in 100 μl serum-free medium were seeded on the upper chamber. Lower chamber was filled with 1640 culture medium supplemented with 2.5% FBS. In comparison group, 5 μl CXCL8 antibody (10 μg/ml) (MAB208, R&D systems) was added in the lower chamber to antagonize the chemotaxis effect of CXCL8, and 2 μl human CXCL8 factor (50 μg/ml) (200-08 M-5UG, PeProTech) was added in the lower chamber of the SKLU-DACH1 and A549-DACH1 group to restore the inhibitory action of DACH1. After incubation for 12 h at 37 °C with 5% CO_2_, migratory cells were stained with 0.5% crystal violet solution and counted by light microscopy (200×).

### Nude mice study

The animal experiment protocols were approved by the ethics committee of the Tongji Hospital of Huazhong University of Science and Technology. The methods used in this section were in accordance with the relevant guidelines and regulations. 1 × 10^5^ A549-vector, A549-DACH1, and A549-ΔDS cells were subcutaneously injected into 4-week-old athymic female nude mice purchased from Hunan SJA Laboratory Animal Co. The tumor volume was measured weekly for 6 weeks by using a digital caliper. Nude mice were sacrificed and subcutaneous tumor lumps were stripped on day 42 after implantation. Tumors were weighed by electronical scale.

### Plasmid construction, transient transfection, and luciferase reporter gene assay

The 2000 bp sequence of CXCL8 promoter was cloned into pGL3-basic vector. The recombinant plasmid consisting of 163 bp sequence of CXCL8 promoter is a gift from Dr. Antonella Casola [33]. Cells were plated at a density of 1 × 10^5^ cells in a 24-well plate on the day prior to transfection with Superfect according to the manufacturer’s protocol (Qiagen, Valencia, CA). Transient transfection with the expression vectors encoding DACH1 and ΔDS were previously described [11, 21, 34]. One hundred fifty nanograms of expression vectors and 200 ng of promoter reporter plasmids were required in each well in accordance with the dose-response fashion. Luciferase reporter gene assay was conducted by the kit (TransDetect, FR101). Fluorescence intensity was quantified with luminometer (Biotech Synergy 2).

GIPZ Lentivirus shRNA vector expressing control or DACH1 was purchased from Open Biosystems. shDACH1-targeted sequences are CTGAAGCAATGAAGGTGAA and TGAGAATGTTTGTAAATGT. Stable cell line HBE was established by infecting with lentivirus expressing shDACH1 or shCTL. H460 cells were planted on an 8-well chamber and were transiently transfected with shDACH1 or shCTL using Lipofectamine 2000 following the standard protocol [19]. After 48 h culture, cells were routinely fixed, permeabalized by 0.5% Triton X-100 and blocked with 5% BSA, and then stained with antibody to DACH1 or CXCL8. Nuclei were count stained with Hoechst 33342.

### Kaplan-Meier plotter

Kaplan-Meier survival curves with hazard ratio and log-rank *p* value were calculated and plotted with the analysis tool, which can be accessed online at http://kmplot.com/analysis/ [35]. The background database, downloaded from GEO (Affymetrix microarrays only), EGA, and TCGA, offers gene expression data, relapse free survival (RFS), and overall survival (OS) rates. The software is capable to assess the effect of 54,675 genes on survival using 10,188 cancer samples, among which includes 2437 cases of lung cancer. We analyzed the survival outcomes of NSCLC and ADC in different expression levels of DACH1 and CXCL8. The Kaplan-Meier survival curves downloaded from the website were resized in Adobe Illustrator CS6. The blend curves were obtained from IBM SPSS Statistics 19.0 using GSE31210 and resized in Adobe Illustrator CS6.

### Statistical analysis

Statistical analyses between groups were calculated by Student’s *t* test and one-way ANOVA. The *p* value was set at 0.05. Mann-Whitney test was applied when the original data did not accord with normal distribution. Correlations between clinic-pathological features and IHC variables were calculated via Pearson chi-square test and continuity correction chi-square test. Based on the extracted survival data of IHC chip, the survival curve for OS and RFS were calculated using Kaplan-Meier method with the log-rank test, and univariate cumulative survival analyses were conducted with Cox regression model. Statistical analyses were conducted using SPSS 19.0 and GraphPad Prism 5.0. All data were presented as the mean ± SEM.

## Results

### CXCL8 mRNA level positively correlates to the progression of ADC

**Fig. 1:**
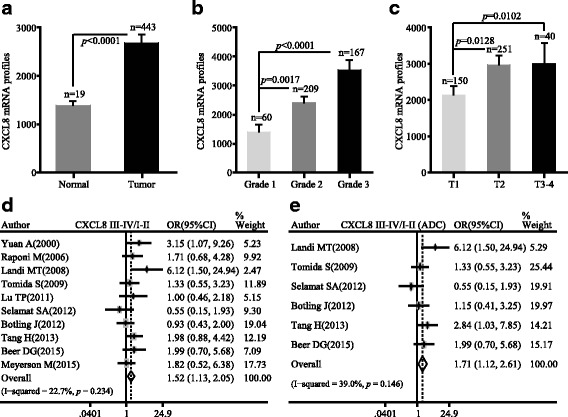
The mRNA profile of CXCL8 was positively correlated with the progression of ADC. **a** CXCL8 mRNA level was higher in ADC patients in comparison with normal tissues. **b** mRNA expression of CXCL8 was in relation to the grade of ADC. **c** mRNA expression of CXCL8 was in relation to the TNM stage of ADC. **d** The forest plot of relative mRNA expression of CXCL8 between III–IV and I–II patients in NSCLC. **e**. The forest plot of relative mRNA expression of CXCL8 between III–IV and I–II patients diagnosed with ADC

Gene expression dataset (GSE68465) was extracted to estimate the mRNA level of CXCL8 in ADC. The results showed that the mRNA profile of CXCL8 was dramatically higher in tumor tissue than normal tissue (*p* < 0.0001) (Fig. 1a). ADC patients with poor and moderate differentiation expressed more CXCL8 than patients with well differentiation (*p* < 0.0001 and *p* = 0.0017) (Fig. 1b). Besides, CXCL8 level showed an upward trend with the increase of tumor size (*p* = 0.0128 and *p* = 0.0102) (Fig. 1c). We further integrated 10 studies consisting of 1070 patients with detailed clinic-pathological characteristics (Table 1). The meta-analysis indicated that increased CXCL8 mRNA level was significantly associated with advanced tumor stage in NSCLC patients (pooled OR = 1.52, 95% CI 1.13–2.05), especially in ADC patients (pooled OR = 1.71, 95% CI 1.12–2.61) (Fig. 1d, e). It was indicated that overexpression of CXCL8 mRNA occurred in ADC patients and was markedly correlated with the progression of ADC.

**Table 1:** Characteristics of studies included for meta-analysis

First author	Year	Duration (months)	Histology	Stage	Patient number	Quality score	Detection	Platform
Yuan A [40]	2000	28	NSCLC	I–III	58	8	Microarray	Perkin-Elmer Applied Biosystems
Tang H [41]	2013	120	NSCLC	I–III	176	9	Microarray	IlluminaHumanWG-6v3.0
Raponi M [42]	2006	144	SQC	I–III	129	8	Microarray	AffymetrixHgu133plus2.0
Tomida S [43]	2009	109.8	ADC	I–III	117	9	Microarray	AgilentWholeHumanGenomeMicroarray 4x44K G4112F
Botling J [44]	2013	120	NSCLC	I–IV	196	8	Microarray	AffymetrixHgu133plus2.0
Beer DG [45]	2002	110.6	ADC	I–III	86	8	Microarray	AffymetrixHumanFullLength HuGeneFL
Landi MT [46]	2008	NR	ADC	I–IV	74	9	Microarray	AffymetrixHgu133a
Lu T [47]	2010	NR	ADC	I–IV	60	8	Microarray	AffymetrixHgu133plus2.0
Selamat SA [48]	2012	NR	ADC	I–III	58	8	Microarray	IlluminaHumanWG-6v3.0 expression
Meyerson M [49]	2015	NR	SQC	I–IV	135	8	Microarray	AffymetrixHgu133a

Cutoff value: median expression

*NR* not reporting, *ADC* lung adenocarcinoma, *SQC* lung squamous cell carcinoma

### Protein abundance of CXCL8 correlates to the progression and prognosis of ADC

**Fig. 2:**
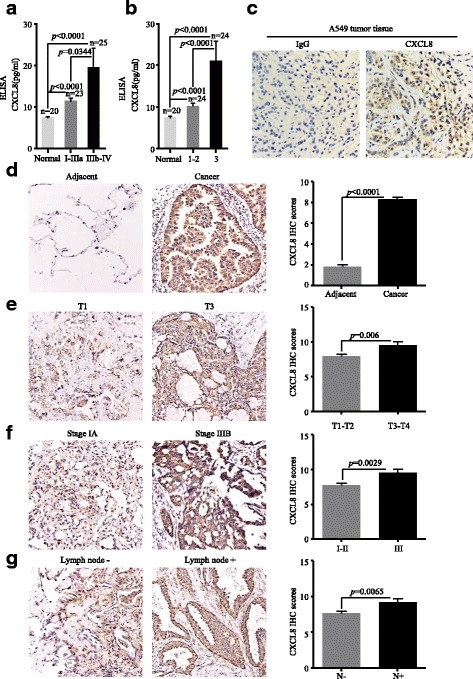
The protein abundance of CXCL8 was positively correlated with the progression of ADC patients. **a** CXCL8 in the serum of ADC patients was associated with the TNM stage. **b** CXCL8 in the serum of ADC patients was associated with the tumor grade. **c** Representative images (200×) of non-specific IgG control and CXCL8 immunohistochemical staining of A549 tumor tissue. **d** Representative images (200×) and quantitative graph of CXCL8 immunohistochemical staining in ADC and corresponding adjacent tissues. **e** Representative images (200×) and quantitative graph of CXCL8 immunohistochemical staining in different tumor sizes. **f** Representative images (200×) and quantitative graph of CXCL8 immunohistochemical staining in different tumor stages. **g** Representative images (200×) and quantitative graph of CXCL8 immunohistochemical staining in ADC with and without lymph node metastasis

In order to determine the protein level of CXCL8, we conducted ELISA to analyze the serum level of CXCL8 in 20 normal samples and 48 ADC patients covering distinct pathological stages and histological grades. Of 48 ADC patients, 23 belong to operable stage I–IIIa, while the rest are unresectable advanced ADC (stage IIIb–IV). The results showed that ADC patients had much higher serum level of CXCL8 than normal samples (I–IIIa/normal: *p* < 0.0001; IIIb–IV/normal: *p* < 0.0001) (Fig. 2a). Moreover, patients with advanced ADC stages secreted more CXCL8 to peripheral circulation than patients with early stage of ADC (*p* = 0.0344) (Fig. 2a). We further analyzed the data based on distinct tumor grades. Patients with grade 3 had highest level of CXCL8 (grade 3/grade 1–2: *p* < 0.0001; grade 3/normal: *p* < 0.0001) (Fig. 2b), and patients with grade 1–2 expressed higher CXCL8 protein level than normal samples (grade 1–2/normal: *p* < 0.0001) (Fig. 2b). Correlation between CXCL8 expression and clinic-pathological features of 48 ADC patients was shown in Table 2.

**Table 2:** Correlations between CXCL8 expression and clinic-pathological features of 68 ADC patients in ELISA

Variables	*N*	CXCL8 expression		*P* value
		Mean (pg/ml)	SEM (pg/ml)	
Sample				< 0.0001^b^
Normal	20	7.45	0.15	
Tumor	48	15.58	2.49	
Age				0.3006^a^
≤ 55	20	11.94	0.77	
> 55	28	18.18	4.20	
Sex				0.4800^a^
Male	29	13.96	1.19	
Female	19	18.06	6.08	
Stage				0.0344^a^
I~IIIa	23	11.35	0.73	
IIIb~IV	25	19.47	4.65	
Grade				< 0.0001^a^
1~ 2	24	10.13	0.52	
3	24	21.03	4.75	

^a^Mann-Whitney

^b^Student’s *t* test

**Fig. 3:**
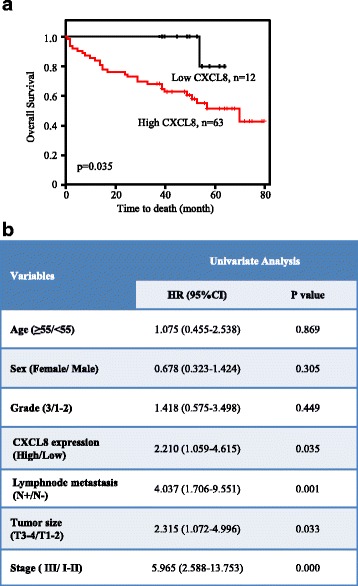
CXCL8 was an independent prognostic factor of OS in ADC patients. **a** Kaplan-Meier survival curve of CXCL8 based on the survival data in tissue microarray slide. **b** Univariate analysis between cumulative OS rates and clinic-pathological variables of ADC patients

Then, we detected the CXCL8 expression on tumor tissues by IHC. Xenograft tumor tissue from A549 cell was used to test the specificity of anti-CXCL8 antibody (Fig. 2c). Immunohistochemical microarray including 75 pairs of ADC patients with survival data, clinic-pathological parameters, and corresponding adjacent tissues was stained and showed that the expression of CXCL8 was markedly higher in ADC tissues than in adjacent normal samples (*p* < 0.0001) (Fig. 2d). Patients with big tumor size (T3-T4) had upregulated CXCL8 expression compared with patients with small tumor size (T1-T2) (*p* = 0.006) (Fig. 2e). The same trend was also found in ADC patients with different TNM stages, that is samples with advanced stage (III) showed stronger staining than samples with early stages (I–II) (*p* = 0.0029) (Fig. 2f). Moreover, ADC patients with lymph node metastasis expressed more CXCL8 than those without lymph node metastasis (*p* = 0.0065) (Fig. 2g). However, there was no statistical difference of CXCL8 expression between tumors with distinct grades. Median OS times of patients with low CXCL8 and high CXCL8 were 51.5 ± 2.52 and 41 ± 2.81 months, respectively (*p* = 0.035) (Fig. 3a). By exploring the relationship between clinic-pathological parameters (age, sex, TNM stage, grade, lymph node metastasis, and tumor size) and CXCL8 expression, we found that the level of CXCL8 was relevant to tumor stage (Pearson chi-square test, *p* = 0.024) and lymph node metastasis (Pearson chi-square test, *p* = 0.004), nevertheless other parameters showed no statistical significance with CXCL8 (Table 3). Univariate Cox regression analysis was used to investigate the correlation between cumulative OS rates and clinic-pathological factors. As shown in Fig. 3b, four factors, including lymph node metastasis (hazard rate (HR) = 4.037; 95% CI 1.706–9.551; *p* = 0.001), tumor size (HR = 2.315; 95% CI 1.072–4.996; *p* = 0.033), TNM stage (HR = 5.965; 95% CI 2.588–13.753; *p* = 0.000), and CXCL8 expression (HR = 2.210; 95% CI 1.059–4.615; *p* = 0.035), were obviously associated with the clinical outcomes of ADC patients, while other parameters were not the direct prognostic factors. Our results suggested that increased CXCL8 protein occurred in ADC patients and was significantly correlated with tumor progression and poor prognosis.

**Table 3:** Correlations between CXCL8 expression and clinic-pathological features of 75 ADC patients in immunohistochemistry chip (HLug-Ade150Sur-02)

Variables	N	CXCL8 expression		*P* value
		≤ 8 (low expression)	> 8 (high expression)	
Age				0.865^a^
< 55	20	14	6	
≥ 55	53	36	17	
Sex				0.255^a^
Male	40	30	10	
Female	35	22	13	
Grade				0.249^b^
1~ 2	61	40	21	
3	14	12	2	
Stage				0.024^a^
I~II	40	30	10	
III	18	8	10	
Lymph node				
N−	36	29	7	0.004^a^
N+	21	9	12	
Tumor size				
T1–T2	60	45	15	0.069^b^
T3–T4	15	7	8	

^a^Pearson chi-square test

^b^Continuity correction chi-square test

### Negative correlation between CXCL8 and DACH1 in vitro

**Fig. 4:**
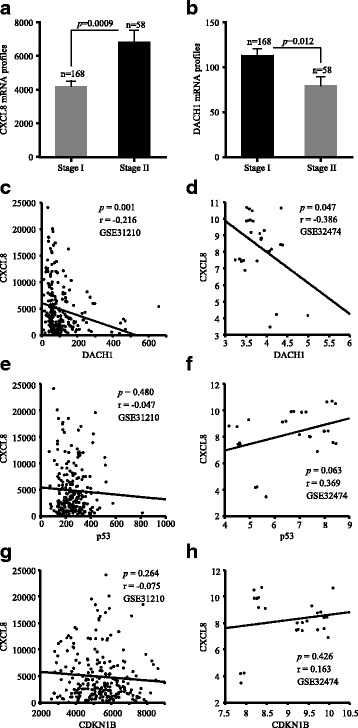
The inverse relationship between CXCL8 and DACH1 at the mRNA level. **a** CXCL8 mRNA profiles in stage I and stage II ADC patients. **b** DACH1 mRNA profiles in stage I and stage II ADC patients. **c** The reciprocal mRNA expression between CXCL8 and DACH1 in ADC tissues from public dataset GSE31210. **d** The reciprocal mRNA expression between CXCL8 and DACH1 in lung cancer cell lines from public dataset GSE32474. **e** The reciprocal mRNA expression between CXCL8 and p53 in ADC tissues from public dataset GSE31210. **f** The reciprocal mRNA expression between CXCL8 and p53 in lung cancer cell lines from public dataset GSE32474. **g** The reciprocal mRNA expression between CXCL8 and CDKN1B in ADC tissues from public dataset GSE31210. **h** The reciprocal mRNA expression between CXCL8 and CDKN1B in ADC tissues from public dataset GSE32474

We analyzed mRNA expression of CXCL8 and DACH1 in early stage of ADC using the Arrayexpress database containing 226 ADC cases with stage I–II (GSE31210). The result showed that CXCL8 mRNA expression was positively correlated with tumor stage at mRNA level (*p* = 0.0009) (Fig. 4a). Meanwhile, DACH1 was negatively correlated with tumor stage in early stage of ADC (*p* = 0.0001) (Fig. 4b). Based on this observation, we conducted relevance analysis between CXCL8 and DACH1 at mRNA level in lung cancer tissues and cell lines. There was a negative correlation between CXCL8 and DACH1 (*p* = 0.001, *p* = 0.047) (Fig. 4c, d). Meanwhile, we also did the correlation analysis between CXCL8 and other known tumor suppressor genes, such as p53 and CDKN1B, and there were no significant negative relationship between CXCL8 and those genes in both lung cancer patients (*p* = 0.480, *p* = 0.264) and lung cancer cell lines (*p* = 0.063, *p* = 0.426) (Fig. 4e–h). It suggested the specificity of correlation between DACH1 and CXCL8 in lung cancer.

**Fig. 5:**
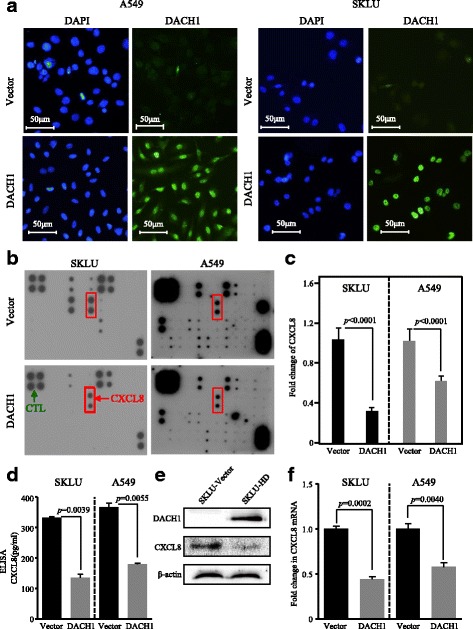
Overexpression of DACH1 repressed the CXCL8 level in vitro. **a** A549 and SKLU cells stably expressing DACH1 were confirmed by immunofluorescence assay. **b** Overexpression of DACH1 intuitively reduced the CXCL8 secreted by SKLU and A 549 cell lines, and **c** the quantitative graph showed marked statistical significance. **d** The protein level of CXCL8 in the supernatant of SKLU-DACH1 and A549-DACH1 cells was significantly lower compared with SKLU-vector and SKLU-vector cells. **e** The protein abundance of CXCL8 was repressed in SKLU cells stably expressing DACH1. **f** The mRNA level of CXCL8 was restrained in SKLU and A549 cells steadily expressing DACH1

**Fig. 6:**
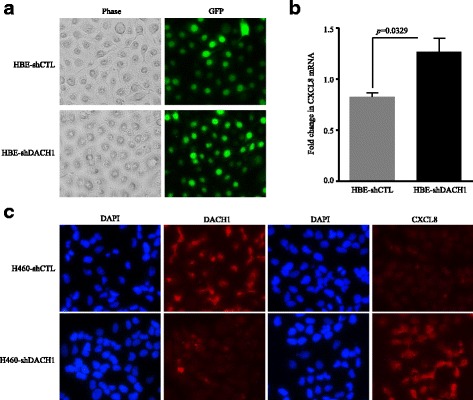
Knockdown of DACH1 upregulated the CXCL8 expression. **a** HBE cells stably expressing shDACH1 or shCTL were confirmed by immunofluorescence assay. **b** The mRNA level of CXCL8 was upregulated in HBE-shDACH1 cells. **c** CXCL8 was significantly increased in the absence of DACH1 in H460 cells

Stable expression of DACH1 in A549 and SKLU cells was confirmed by immunofluorescence assay (Fig. 5a). Human cytokine array analysis intuitively showed that SKLU-DACH1 and A549-DACH1 cells secreted lower level of related cytokines, especially CXCL8, compared with the vector control group (Fig. 5b). Compared with the control group which was standardized as 1, the confidence intervals of CXCL8 expression level were 0.2–0.4 in SKLU-DACH1 cells (*p* < 0.0001) and 0.5–0.7 in A549-DACH1 cells (*p* < 0.0001). And the corresponding quantitative bar graph certified the trend (*p* < 0.0001) (Fig. 5c). Similarly, cell lines ELISA showed that lower protein levels of CXCL8 were detected in the conditional medium of SKLU-DACH1 and A549-DACH1 cells than in the SKLU-vector and A549-vector cells (*p* = 0.0039, *p* = 0.0055) (Fig. 5d). Western blot further suggested that SKLU-DACH1 cell line expressed decreased CXCL8 protein (Fig. 5e). We also conducted the real-time PCR to explore the mRNA level of CXCL8 using SKLU-vector, SKLU-DACH1, A549-vector, and A549-DACH1 cells. The results certified that CXCL8 mRNA expression was significantly downregulated under the condition of the existence of DACH1 regarding the corresponding vector group as the criterion (*p* = 0.0002, *p* = 0.004) (Fig. 5f). HBE cells steadily expressing shDACH1 and shCTL were established, as evidenced by GFP expression from vector (Fig. 6a). Real-time PCR analyses showed that the mRNA level of CXCL8 was upregulated in the loss of DACH1 (*p* = 0.0329) (Fig. 6b). Immunofluorescence assay exhibited the consistent trend that the inhibition of DACH1 could markedly stimulate the CXCL8 expression in H460 cell (Fig. 6c). Overall, we certified that DACH1 was a vital factor to inversely regulate the CXCL8 in vitro.

### DACH1 represses CXCL8-induced migration of lung cancer cells in vitro

**Fig. 7:**
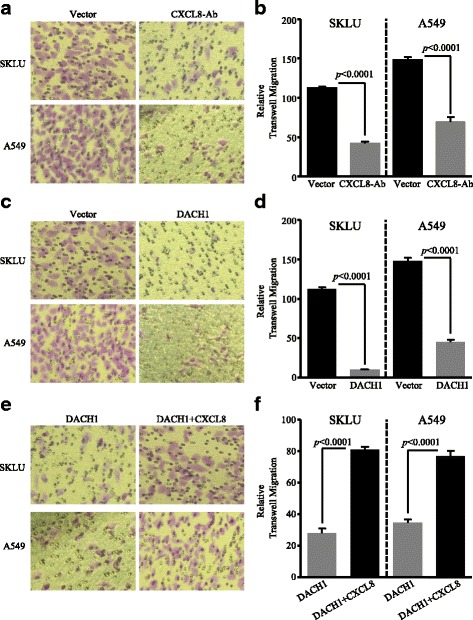
Overexpression of DACH1 controlled the CXCL8-induced migration of lung cancer cell in vitro. **a** Anti-CXCL8 antibody controlled the migration of SKLU and A549 cell lines, and **b** the corresponding quantitative graph showed statistical significance. **c** The migration of ADC cells was inhibited in the presence of excessive DACH1 expression, and **d** the corresponding quantitative graph showed statistical significance. **e** Human CXCL8 cytokine restored the DACH1-induced migration inhibition of SKLU-DACH1 and A549-DACH1 cells, and **f** the corresponding quantitative graph showed statistical significance

To investigate whether overexpression of CXCL8 had a critical role in cell movement of ADC and whether DACH1 could interfere the CXCL8-induced migration, we performed transwell assay using three groups. Anti-CXCL8 antibody efficiently inhibit cell migration in both SKLU and A549 cells in comparison with the vector cells (*p* < 0.0001, *p* < 0.0001) (Fig. 7a, b). Meanwhile, the number of migratory cells was markedly reduced in SKLU and A549 cells expressing DACH1 compared with the vector group (*p* < 0.0001, *p* < 0.0001) (Fig. 7c, d). Based on this, we added the human CXCL8 factor into the SKLU-DACH1 and A549-DACH1 cells, and we found that CXCL8 factor restored the migratory ability of SKLU and A549 in spite of the presence of DACH1 (*p* < 0.0001, *p* < 0.0001) (Fig. 7e, f).

### DACH1 inhibits the ADC tumor growth and is negatively correlated with CXCL8, cyclin D1, and Ki-67 expression in vivo

**Fig. 8:**
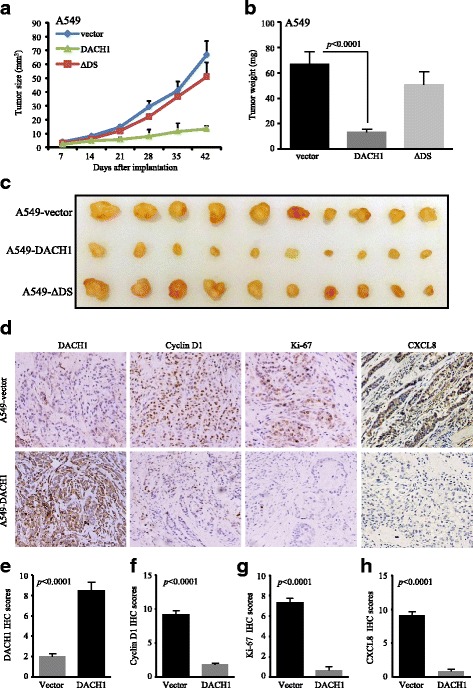
Overexpression of DACH1 inhibited the ADC growth and was negatively correlated with CXCL8, cyclin D1, and Ki-67 expression in vivo. **a**. Tumor growth curve, **b** tumor weight, and **c** representative images of xenograft tumor models implanted with A549-vector, A549-DACH1, and A549-ΔDS cells. **d** Representative images of DACH1, cyclin D1, Ki-67, and CXCL8 immunohistochemical staining from xenograft tumor tissues implanted with A549-vector and A549-DACH1 cells. Corresponding IHC scores of DACH1 (**e**), cyclin D1 (**f**), Ki-67 (**g**), and CXCL8 (**h**) showed obvious statistical significance

The xenograft tumor models were established through subcutaneous injection of A549-vector, A549-DACH1, and A549-ΔDS cells in nude mice (Fig. 8c). A549 cells with sustained DACH1 expression possessed less tumorigenic potential and slower tumor growth rate than A549-vector and A549-ΔDS cells in vivo. Increased DACH1 expression inhibited the volume of tumor by 85% and decelerated the tumor growth (Fig. 8a). Besides, tumor weight was significantly reduced by 85% in mice implanted with A549-DACH1 cells in comparison with the control group (*p* < 0.0001) (Fig. 8b). Nevertheless, no statistical difference was found in the tumor growth rate and final tumor weight between the A549-vector tumor group and A549-ΔDS tumor group (Fig. 8a, b).

Immunohistochemical staining of xenograft tumor tissues was conducted to evaluate the protein abundance of DACH1, cyclin D1, Ki-67, and CXCL8 in DACH1-overexpressed and control tumors. Images intuitively showed negative associations between DACH1 and the expressions of cyclin D1, Ki-67, and CXCL8 (Fig. 8d). And the semi-quantitative IHC scores exhibited the accordant trend that high level of DACH1 prominently downregulated the protein abundance of cyclin D1 (*p* < 0.0001), Ki-67 (*p* < 0.0001), and CXCL8 (*p* < 0.0001) (Fig. 8e–h). Our results suggested that besides inhibiting the CXCL8 expression, DACH1 could also reduce the expression of proliferation-related proteins, such as cyclin D1 and Ki-67, which explained the mechanism by which DACH1 restrained the tumorigenesis and growth processes in nude mice model.

### DACH1 transcriptionally represses CXCL8 through AP-1 and NF-κB binding sites

**Fig. 9:**
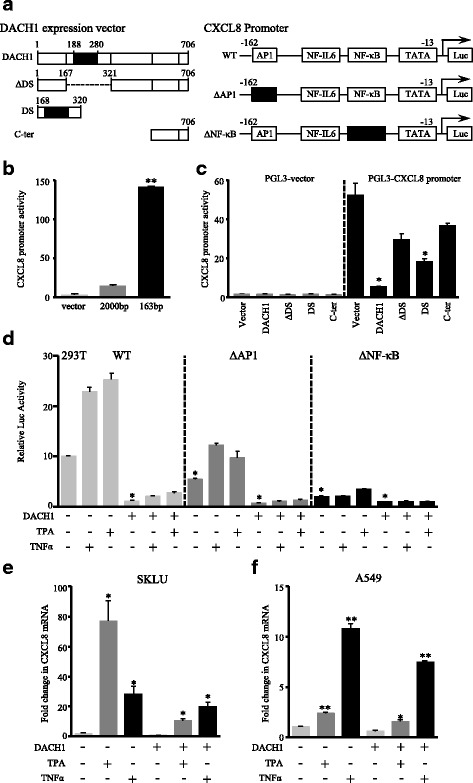
DACH1 repressed CXCL8 expression through binding to AP-1 and NF-κB sites of CXCL8 promoter in the presence of the DS domain. **a** Schematic representation of distinct DACH1 expression vectors and CXCL8 promoter mutants used in luciferase reporter gene assay. **b** The basal activities of empty vectors and plasmids containing different CXCL8 promoters. **c** The CXCL8 promoter activity in 293T cells transfected with different DACH1 expression vectors. **d** Relative luciferase activity of CXCL8 promoter and point mutants in 293T cells with and without steady DACH1 expression. TPA and TNF-α were used to stimulate CXCL8 production. **e** mRNA level of CXCL8 was detected by real-time PCR in SKLU-vector and SKLU-DACH1 cells under the stimulation of TPA and TNF-α. **f** mRNA level of CXCL8 was detected by real-time PCR in A549-vector and A549-DACH1 cells under the stimulation of TPA and TNF-α. * *p* < 0.05; ** *p* < 0.0001

To determine the mechanisms by which DACH1 suppressed the CXCL8 expression, several mutant DACH1 expression plasmids were examined to take effect on the CXCL8 promoter (Fig. 9a). We detected CXCL8 promoter activities under the influence of different DACH1 expression plasmids by using luciferase reporter gene assay. Before any treatment, we detected the basal fluorescence activity of empty vector, plasmids containing 2000 bp sequence of CXCL8 promoter and 163 bp sequence of CXCL8 promoter. As shown in Fig. 9b, plasmid containing 163 bp sequence of CXCL8 promoter exhibited higher basic activity (*p* < 0.0001); thus, we chose it for next experiments. The result suggested that DACH1 repressed the CXCL8 promoter mainly via its DS domain (DACH1/vector: *p* = 0.0016; DS/vector: *p* = 0.0056) (Fig. 9c). Meanwhile, activities of point mutants of CXCL8 promoter were detected under the effect of DACH1. DACH1 inhibited the activity of CXCL8 promoter by 90% (*p* = 0.0002). Mutations of the AP-1 site and NF-κB site on the CXCL8 promoter downregulated CXCL8 promoter activity by 50% and 80% respectively (*p* = 0.0019, *p* = 0.0003) and interfered the inhibitory function of DACH1 to some extent (Fig. 9d). DACH1 repressed ΔAP-1 and ΔNF-κB mutants by 75 and 50% (*p* = 0.0013, *p* = 0.0089) (Fig. 9d).

CXCL8 stimulators, TPA and TNF-α, were supplemented to microenvironment of transfected 293T cells for 12 h. Luciferase reporter gene assay showed that TPA and TNF-α enhanced the CXCL8 promoter activity majorly through AP-1 and NF-κB sites, while the existence of DACH1 repressed the activation of CXCL8 promoter stimulated by TPA and TNF-α (Fig. 9d). Real-time PCR was conducted to evaluate the CXCL8 mRNA level in SKLU-vector, SKLU-DACH1, A549-vector, and A549-DACH1 cells under the influence of TPA and TNF-α. It was showed that TPA and TNF-α substantially stimulate the expression of CXCL8 (Fig. 9e, f). Overall, our results proved that DACH1 transcriptionally suppressed CXCL8 through AP-1 and NF-κB sites of CXCL8 promoter in the dependent of the DS domain of DACH1. TPA and TNF-α, acted as two major CXCL8 stimulators, efficiently upregulated the CXCL8 expression.

### Relative expressions of CXCL8 and DACH1 predict survival in NSCLC

**Fig. 10:**
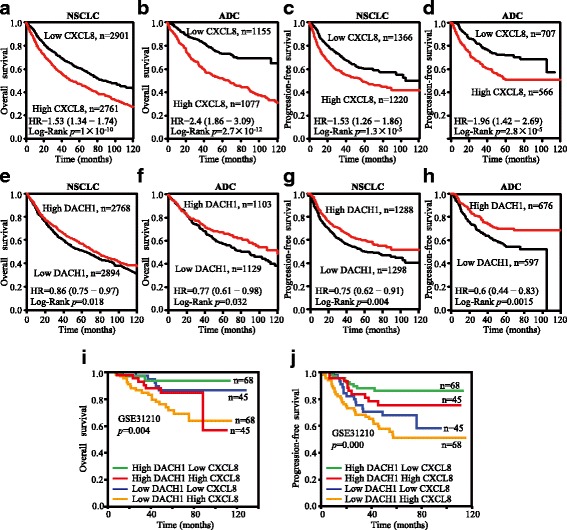
High CXCL8 and low DACH1 expression predicted poor survival time of patients with NSCLC, especially in ADC. **a** Kaplan-Meier survival curves of CXCL8 with OS of NSCLCs. **b** Kaplan-Meier survival curves of CXCL8 with OS of ADCs. **c** Kaplan-Meier survival curves of CXCL8 with PFS of NSCLCs. **d** Kaplan-Meier survival curves of CXCL8 with PFS of ADC. **e** Kaplan-Meier survival curves of DACH1 with OS of NSCLC. **f** Kaplan-Meier survival curves of DACH1 with OS of ADC. **g** Kaplan-Meier survival curves of DACH1 with PFS of NSCLC. **h** Kaplan-Meier survival curves of DACH1 with PFS of ADCs. **i** The blend Kaplan-Meier survival curves of CXCL8 and DACH1 with OS of ADC in GSE31210. **j** The blend Kaplan-Meier survival curves of CXCL8 and DACH1with PFS of ADC in GSE31210

Kaplan-Meier curves indicated that patients with higher mRNA level of CXCL8 had shorter OS time and RFS time, which represent poor survival in NSCLC (Fig. 10a, c). And the subgroup analysis of ADC showed the same trend (Fig. 10b, d). On the contrary, high DACH1 predicted favorable OS and RFS in NSCLC, especially in ADC (Fig. 10e–h). The blend Kaplan-Meier curves showed that patients with high DACH1 and low CXCL8 had the longest OS and RFS time, whereas low DACH1 and high CXCL8 predicted poorest prognosis (Fig. 10i, j). Taken together, our study was consistent with a previous study [25] that DACH1 was negatively correlated with tumor progression, whereas high CXCL8 expression was associated with unfavorable prognosis of NSCLC patients.

## Discussion

Tumor microenvironment plays a pivotal role in sustaining tumor progression. Numerous cytokines, such as CXCL8, IL-6, and CXCL5, trigger inflammatory responses by recruiting and activating relevant inflammation cells in the tumor foci resulting in tumor growth, progression, and immunosuppression [4, 36, 37]. CXCL8 is responsible for the promotion of the long-lasting chronic inflammation, which in turn contributes to the tumorigenesis [4]. Increased CXCL8 is detected in lung cancer, especially in NSCLC cell lines [8]. Elevated CXCL8, acting as a potently proangiogenic factor, mediates neovascularization in coordination with vascular endothelial growth factor (VEGF). Meanwhile, CXCL8 promotes proliferation and induces epithelial-mesenchymal transition (EMT) in lung cancer cells. Together, high CXCL8 is associated with advanced stages and distant lymph node metastasis, contributing to the shortened survival time and early relapse [10, 38]. However, the exact mechanism underlying CXCL8 overexpression in tumor cells is not well defined.

Whole-genome and transcriptome sequencing of NSCLC tumor and normal tissues identified mutation and copy number loss of DACH1 [39]. DACH1 had been proved to be negatively correlated with the development and progression of breast cancer and prostate cancer [17, 21]. In previous study, we found that DACH1 restrained CXCL8-induced invasion and metastasis of breast cancer through selectively interacting with the AP-1 and NF-κB binding sites of the CXCL8 promoter [20]. We are inspired by these studies and conducted relevant mechanism explorations to prove the relationship between DACH1 and CXCL8 in lung cancer. In agreement with previous result, DACH1 transcriptionally downregulates the CXCL8 in NSCLC, and an inverse relationship between CXCL8 and DACH1 was identified in lung cancer cell lines and tumor tissues.

In this study, high expression of CXCL8 was detected in NSCLC both at mRNA and protein levels and was also remarkably associated with poor prognosis of lung cancer patients. Specifically, patients with advanced tumor stage, higher tumor grade, and bigger tumor size exhibit higher CXCL8 mRNA levels. CXCL8 secreted from tumor cells via an autocrine manner acts on tumor microenvironment and enters the peripheral blood. Consequently, patients diagnosed as ADC showed elevated protein abundance of CXCL8 in tumor tissues and the peripheral circulation. We found that CXCL8 protein was positively correlated with high tumor burden, including lymph node metastasis, advanced TNM stage, big tumor size, and poor OS time. CXCL8 is a dominant factor stimulating the growth, metastasis, and progression of ADC, which also can act as a monitoring index to assess the clinical outcomes of ADC.

Meanwhile, our group revealed the potential role of CXCL5, another member of C-X-C family, in the progression and prognosis of NSCLC [6]. It was reported that DACH1 was negatively associated with CXCL5 and could inhibit invasion and growth of ADC through restraining CXCL5 signaling [25]. In this study, we identified the reverse relationship between DACH1 and CXCL8 in ADC both in vitro and in vivo. Experimental studies in ADC cell lines (A549 and SKLU) steadily expressing DACH1 showed that CXCL8 was downregulated at both mRNA and protein level. On the other hand, the loss of DACH1 could abolish its suppressive effect on CXCL8 and lead to hyperactivation of CXCL8. Functionally, DACH1 significantly suppressed CXCL8-induced migration of A549 and SKLU cells, which indicated that the cancer-promoting effects of CXCL8 could be attenuated by the expression of DACH1 in ADC. Based on the results of the ADC xenograft mice model, DACH1 efficiently inhibited the expression of CXCL8 and the pro-proliferative factors, such as cyclin D1 and Ki-67, which in turn significantly retarded the tumor growth. Collectively, CXCL8 is a potential target of DACH1, and DACH1 could repress the expression and function of CXCL8 mediating NSCLC cellular growth and metastasis in vitro and in vivo.

Two main transcriptional activators on CXCL8 promoter are AP-1 and NF-κB. TNF-α and TPA effectively promoted CXCL8 activation through interacting with different sites. DACH1 could markedly repress the basal level activity of CXCL8 and the stimulatory role of TNF-α and TPA. Further analysis revealed that DS domain was required in the above process, which is consistent with the previous research [20].

## Conclusions

This study confirms the relationship between CXCL8 and clinic-pathological parameters of ADC. CXCL8 could be an independent prognostic factor of OS and RFS in ADC patients. DACH1 was negatively related to CXCL8, and restoration of DACH1 antagonizes CXCL8. Our study indicated that DACH1 was a key factor to inversely regulate CXCL8. Double detection of DACH1 and CXCL8 could provide precise information for predicting the prognosis of ADC patients.

## Abbreviations

ADC: Lung adenocarcinoma
AP-1: Activator protein-1
cDNA: Complementary DNA
CI: Confidence interval
CO_2_: Carbon dioxide
CXCL8: C-X-C motif ligand 8
DACH1: Dachshund homologue 1
DMEM: Dulbecco’s modified Eagle’s medium
DTT: DL-Dithiothreitol
EGFR: Epidermal growth factor receptor
ELISA: Enzyme-linked immunosorbent assay
ELR: Glutamic acid-leucine-arginine
EMT: Epithelial-mesenchymal transition
ER-α: Estrogen receptor-alpha
FBS: Fetal bovine serum
HBE: Human bronchial epithelial
HR: Hazard rate
IHC: Immunohistochemistry
IL-1β: Interleukin-1β
IL-8: Interleukin-8
NF-κB: Nuclear transcription factor-kappa B
NSCLC: Non-small cell lung cancer
OD: Optical densities
OS: Overall survival
PBS: Phosphate buffered solution
PMSF: Phenylmethanesulfonyl fluoride
PRX3: Peroxiredoxin 3
PVDF: Polyvinylidene fluoride
RFS: Relapsed-free survival
SDS: Sodium dodecyl sulfate
TGF-β: Transforming growth factor-beta
TNF-α: Tumor necrosis factor-α
TPA: 12-O-tetradecanoyl phorbol-13-acetate
VEGF: Vascular endothelial growth factor
