# Correction: DACH1 antagonizes CXCL8 to repress tumorigenesis of lung adenocarcinoma and improve prognosis

**DOI:** 10.1186/s13045-022-01393-2

**Published:** 2022-12-13

**Authors:** Qian Liu, Anping Li, Shengnan Yu, Shuang Qin, Na Han, Richard G. Pestell, Xinwei Han, Kongming Wu

**Affiliations:** 1 Department of Oncology, Tongji Hospital of Tongji Medical College, Huazhong University of Science and Technology, Wuhan, 430030 People’s Republic of China; 2 Department of Interventional Radiology, The First Affiliated Hospital of Zhengzhou University, Zhengzhou, 450052 People’s Republic of China; 3 Pennsylvania Cancer and Regenerative Medicine Research Center, Wynnewood, PA 19096 USA

**Correction: Journal of Hematology & Oncology (2018) 11:53** 10.1186/s13045-018-0597-1

The original article contained errors in Fig. 7. A549 vector in panel **a** should be labelled as vehicle and was duplicated with A549 vector in panel **c**. SKLU DACH1 in panel **e** should be labelled SKLU DACH1-vehicle, and was duplicated with SKLU CXCL-Ab in panel **a**. We repeated this experiment and presented right images with corresponding analysis.

**Fig. 7:**
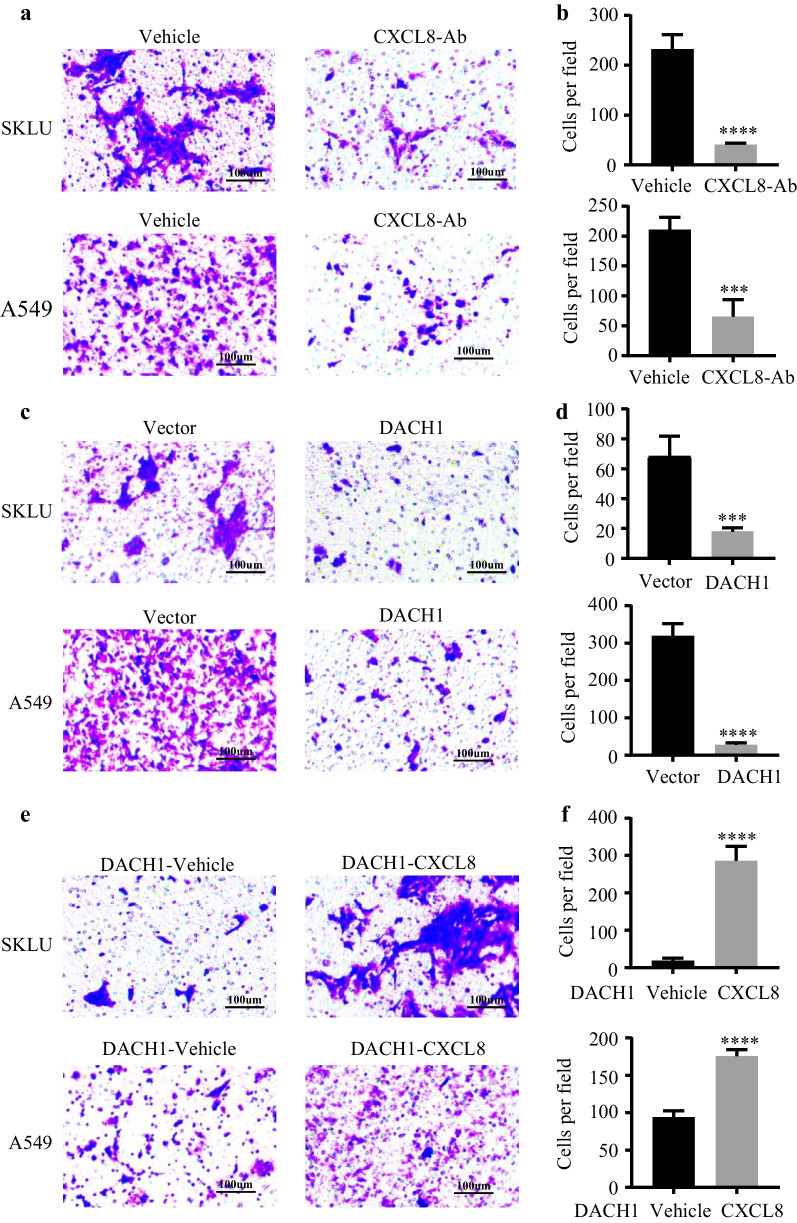
Overexpression of DACH1 controlled the CXCL8-induced migration of lung cancer cell in vitro. **a** Anti-CXCL8 antibody controlled the migration of SKLU and A549 cell lines, **b** the corresponding quantitative graph showed statistical significance. **c** The migration of ADC cells was inhibited in the presence of excessive DACH1 expression, **d** the corresponding quantitative graph showed statistical significance. **e** Human CXCL8 cytokine restored the DACH1-induced migration inhibition of SKLU-DACH1 and A549-DACH1 cells, **f** the corresponding quantitative graph showed statistical significance

